# A Treatise on Lightning Conductors; Compiled from a Work on Thunderstorms

**Published:** 1853-08

**Authors:** 


### REVIEWS.

---

ART. V.—A *Treatise on Lightning Conductors;* compiled from a work on Thunderstorms, by S. W. HARRIS, F. R. S., and other standard authors. By LUCIUS LYON, A. M. New York: George P. Putnam. 1853.

*(Concluded.)*

In a notice of this valuable work, in the July number of this Journal, we endeavored to develope some of the curious facts connected with electrical phenomena. We pass now to the principles concerned in the metallic conductors. The remarks on these laws are invaluable, and we make liberal extracts. The author says:

"The heating effect of the same, or different quantities of electricity, on metallic wires of the same or different diameters, is as the square of the passing quantity of electricity directly, and as the square of the diameter of the wire inversely. Thus the heat developed by a passing shock is four times as great when the quantity of electricity is doubled, and only one-fourth as great when the diameter of the wire is doubled. A metallic rod, therefore, of twice the diameter, conducts twice the quantity of electricity with the same development of heat. Taking the heat evolved as proportionate to the resistance, we may conclude, that the conducting power of a metallic body varies with the area exposed in cutting it transversly to its length; that is to say, with the area of its section, since this is proportionate to its solid contents.

In estimating the resistance of a metallic body to the transmission of a shock of electricity, we have also to take into consideration the distance traversed. Now the resistance to electrical transmission through a metallic wire, has been found to increase with the length of the wire; that is to say, the resistance to the transmission of the charge was twice as great when the length of the conductor was doubled; a law observable from one hundred up to one thousand feet in length. A lightning conductor, therefore, should have its dimensions increased, when required to be of considerable extent.

The explanation of the above general laws seems to be this: supposing a given quantity of electricity to fall on a single metallic particle, and to experience a given resistance to its progress, then this resistance would be diminished by placing a second particle by the side of it, for the charge would be divided between the two.—If two other particles were added, we may conceive it to be still further reduced in proportion to the number of particles sharing in the conduction. Now, in the case of the section of a metallic wire, the diameter of which is double that of another, there are four times the number of particles,—hence the resistance with the same quantity of charge is reduced to one-fourth. In a similar way it may be shown, that by increasing the length of the conductor, we continually increase the number of particles to be passed through,—hence the resistance through twice the length will be twice as great, through three times the length three times as great, and so on. In the case of the quantity of electricity being increased, we have the resistance dependant not only on the increased charge, but also on its increased force. Thus a particle of metal conducting a double quantity of electricity is subject to a double force, since it is quite reasonable to conclude that the forces with which the opposite electrical powers tend to unite, would increase with the amount of disturbance.

*Mechanical Effects of the Electrical Discharge.*—But it is not only the heating effect of the discharge we have to consider; it is necessary to take also into the account its more mechanical effects,—indeed, it is the expansive action which produces the great mass of damage by lightning so commonly observed. If a powerful shock of electricity be transmitted by a fine wire, the wire will very often appear crippled throughout its length, and will exhibit a series of zigzag creases. And if a similar shock be passed between two metallic balls in a confined portion of air, the air will be caused to expand with great violence, so as to frequently burst open the containing vessel. Light bodies such as wafers will become dispersed in all directions, when exposed to the expansive effect produced by the electric shock in passing through a short interval of air.

At the time the steeple of St. Bride's church, London, was struck by lightning, in June, 1764, it is stated by Mr. Delaval, F. R. S., who communicated a most interesting detail of the circumstances to the Royal Society, that "the lightning acted as an elastic fluid," and that "the effects are exactly similar to those which would have been produced by gunpowder, pent up in the same places and exploded. A stone weighing about seventy pounds was thrown to a distance of fifty yards; an iron bar half an inch in thickness, and about two feet in length, joining some of the masonry of the tower, was not only broken in two, but one part of it, according to Dr. Watson,* was actually bent to an angle of 45°."

The mechanical effects of lightning, seen in piercing solid bodies with holes, in splitting them in pieces, and in projecting their fragments (sometimes of enormous weight) to great distances, are so well known, and so generally admitted, that it will be needless to multiply instances in proof of it; but a circumstantial statement of some remarkable cases of this kind may throw light upon the manner in which the electric fluid acts.

In the autumn of 1798, lightning struck the house of Casselli, an Engineer, at Alexandria. It did no damage, but pierced the panes of glass in the windows with several small holes about the sixth of an inch in diameter. Small cracks in the glass diverged from these holes as centres.

In August, 1777, lightning struck the steeple of the parish church of St. Sepulchre at Cremona, broke the

---

* *Phil. Trans.* for 1764.

iron cross which surmounted the tower, and projected to a distance the weathercock, which revolved under the cross, and which was made of copper, tinned, and coated with oil-paint.

This weathercock was found to have been pierced with *eighteen holes*, nine of which were very prominent on one side, and the other nine on the other. As there was no appearance of more than one stroke of lightning, all these holes must be supposed to have been pierced at once. The position of the holes is such as would have been produced by blows imparted simultaneously in opposite directions on parts of the metal nearly contiguous, and the inclination of the beards or projecting edges of the holes on one side correspond exactly with those on the other, the directions of all the eighteen beards being parallel.

On the 3d of July, 1821, lightning struck a house at Geneva, and pierced the tin which covered a part of the roof with several holes, leaving evident marks of fusion. One piece of tin in particular, which covered the angle made by a chimney with the surface of the roof near it, was pierced with three nearly circular holes, about an inch and three quarters in diameter, and about five inches apart measured from centre to centre. The metal at the edges of these holes was bent, as it would have been by a force bursting through it in one direction or the other. The edges of the two holes were bent on contrary sides.

On the night between the 14th and 15th of April, 1718, the church of Gouesnon, near Brest, was struck by lightning with such force that it shook as if by an earthquake. The stones of the walls were projected in all directions to a distance of from fifty to sixty yards.

The lightning which formerly struck the chateau of Clermont, in Beauvoisis, made a hole twenty-six inches wide and the same depth in the wall; the date of the building of which was so far back as the time of Cæsar, and which was so hard that a pickaxe could with difficulty make any impression upon it.

On the night between the 21st and 22d of June, 1723, lightning struck a tree in the forest of Nemours. The trunk was split into two fragments, one seventeen, and the other twenty-two feet long. These fragments, so heavy that one of them would require the combined strength of four men, and the other that of eight men, to lift it, were, nevertheless, projected by the lightning to the distance of about seventeen yards.

In January, 1762, lightning struck the church of Breag, in Cornwall, the southwest pinnacle of the tower of which it destroyed. A stone, weighing one hundred and seventy pounds, was projected from the roof of the church to a distance of sixty yards in the direction of the south. Another fragment of stone was projected to the north to a distance of 400 yards. A third was projected to the southwest.

About the middle of the last century, a rock of micaceous schist, measuring 105 feet long, 10 feet wide, and about four feet thick, was struck by lightning at Funzie, in Scotland, and was broken into three principal fragments, not counting smaller pieces. One of these fragments twenty-six feet long, ten feet wide, and four feet thick, had been merely inverted in its position. Another, 28 feet long, 7 feet wide, and five feet thick, was projected over the hill to a distance of fifty yards. The remaining piece, forty feet long, was projected in the same direction, with still greater force, and fell into the sea.

On the 6th of August, 1809, at Swinton, about five miles from Manchester, lightning struck the house of Mr. Chadwick, at 2 P. M. A sulphurous vapor immediately filled the house. The external wall of a building erected against the house as a coal-shed, was torn from its foundations, and raised in a mass. It was transported, maintaining its vertical position, to some distance from its original place ; one of its ends was transported nine and the other four feet. This wall thus raised and transported, was composed of seven thousand bricks, which, independent of the mortar by which they were cemented together, would have weighed about twenty-six tons. This wall was eleven feet high and three feet thick, and its foundation was about a foot below the level of the ground. Above this coal-shed was a cistern, which, at the time of the phenomenon, contained a quantity of water, and the shed contained about a ton of coals.*

The first Presbyterian church in the city of Syracuse, New York, was struck by lightning. The church had

---

* Dr. Lardner's Lect. on Science and Art, vol. ii. p. 69.

lightnining rods attached, but these were improperly fastened to the walls by sharp pointed staples in full connection with the main rod. The lightning struck the spire, ran down part way, when the charge divided, following a staple fastening into the building, as far as the pointed staple extended. This staple terminating in the dry, hard masonry which must have been nearly a non-conductor, the charge blasted out a por ion of the steeple,—throwing the broken fragments several hundred feet much after the manner of a too shallow blast in a rock. And thus the beautiful structure was saved the dire destruction which must have happened had the church been built of wood, a partial conductor when wet, and fastened with iron bolts, &c. These facts were related to the writer a few months since by several intelligent and respectable citizens of that city who were eye-witnesses to the accident, each of whom saved peices that were thus blasted out of the steeple. The mechanical force here manifested was precisely like that of gunpowder.

In the shock of lightning which fell on His Majesty's ship *Elephant*, of seventy four guns, at Portsmouth, in November, 1790, all the iron hoops and wooldings on the mainmast were burst open and broken in peices, and some of the parts scattered to great distances. Some of these hoops were half an inch thick, and five inches wide; the mast, although of immense size, being about three feet in diameter, and upwards of 110 feet in length, was entirely shook and shivered throughout. Now, in these cases we do not find any melting of the iron work : we observe only the effects of a terrific mechanical power.

Another remarkably interesting instance of this effect was observed in the case of His Majesty's ship *Desirèe*, already alluded to, struck by lightning in Port Antonio, in Jamaica, in the autumn of 1803. Admiral Ross, who then commanded the ship, states, that one part of the main topmast was found on the following morning sticking in the mud on one side of the harbor, and another part in a timber-yard on the opposite side.

In the application of lightning conductors to buildings, therefore, we have to consider the effect likely to be produced on them by the mechanical action of the shock, by which they may be disjointed, twisted or rent asunder in various ways. Thus, the small conductor of linked brass rod, at Charles church, Plymouth, struck by lightning in December, 1824, was literally torn in pieces and disjointed, and many of the links twisted into the shape of the letter S. A part of the small wire rope applied as a conductor to the Hotel des Invalides at Paris was broken into small pieces an inch or more in length, and scattered in all directions by the lightning which fell on that building in June, 1839. This conductor consisted of about twenty iron wires twisted together as a rope; the lead surrounding the lantern was torn up and scattered, but without any signs of fusion.

The intensity of electrical accumulation having been found to decrease in an inverse ratio of the square of the opposed surfaces, some electricians were led to imagine that extent of surface was the great desideratum in the application of a lightning conductor. This decrease of intensity, however, does not affect the conditions of conduction as regards the heating effect of the discharge, for, whether the quantity of electricity be accumulated on a large extent of surface, or on a small one, the heating effect, when the discharge does occur, is always the same. Whatever may be the distance at which the neutralization of the forces begins, they always unite with precisely the same degree of power, the quantity of electricity being the same.

This question, so frequently discussed, has not been fully appreciated in all its details, for although quantity of metal is an essential condition of a lightning rod, yet it is likewise desirable to place the metallic particles under as great an extent of surface as may be consistent with strength and durability, in order to keep down the intensity of the shock, and diminish the mechanical action. We may, in fact, for the moment, consider a conductor, while in the act of carrying off a charge of lightning, as an electrified body. Its electrical intensity, therefore, is very much less with a large surface, than with a small one: hence, by extent of surface, we diminish the activity of the passing charge, and tranquilize its mechanical effect on the conductor. Thus we find, in a variety of cases of damage by lightning, that the passing charge, in striking on large expanded sheets of metal, has become comparatively tranquil, and has been traced no further, whilst in striking on larger masses of metal, exposing but a small surface, it has assumed an intensely active state. The flash of lightning which struck His Majesty's ship *Badger*, lying in the Medway, in August, 1822, vanished, after striking upon the copper lining of the galley, although just before, it had penetrated the mast, rent the copper, and melted the lead over the heads of two large bolts in the deck beams.

Although the general tendency of a discharge of lightning is always through the conductor, as being the line of least resistance, yet there may arise cases in which the electrical forces may be so circumstanced as to affect other lines of action near it, and in which damage may ensue. Thus, in the French frigate *Calypso*, fitted with a wire conductor of small surface, two men in the main chains, who were standing in a slightly interrupted conducting communication between the wire and the sea were struck senseless. A similar oase is related by M. Arago, as having occurred in the Fuench frigate *Junon*. In fact, wherever from any cause the resistance in the direction of the conductor is considerable, the chances of damage in some other direction become multiplied. Thus, a case occurred at Bayonne, in which the length of the conductor was unnecessarily increased,—that is to say, instead of terminating at once in the ground, it was led off at the foot of the building on semi-insulating stakes of wood for some distance; the charge here divided upon other lines near the conductor, and caused damage.

Provided the quantity of metal be present, the form under which we place it is evidently of no consequence to its conducting power, since it would be absurd to suppose that a mass of metal, under any form, did not conduct electricity in all its particles,—indeed, we know that it does so, and that it is impossible to fuse by electricity *a portion only* of a homogeneous metallic plate of uniform thickness. Again, if the heating effect of a given quantity of electricity on a metallic wire be measured, and then the wire be rolled out into a flat surface, or otherwise drawn out and placed under the form of two or more smaller ones, still the same heat will be evolved when conducting the same charge; there is consequently no disadvantage in giving a lightning rod as much superficial capacity as possible as regards conducting power, whilst, on the contrary, the diminished intensity attendant on it is very advantageous: this effect of superficial conductors appears to depend on the removal of the electrical particles further out of the sphere of each other's influence. In a dense solid rod they may be supposed to be in a state of compression, in each other's way, as it were,—whereas by expanding the same quantity of metal into a larger surface, we immediately free them from this condition, and allow them greater space.

In order, therefore, to resist the heating effect, we require quantity of metal; to restrain the electrical intensity, and to diminish the mechanical force, we require extent of surface. The distinction is nice but it is a very important one."

Important questions naturally spring from these facts. One of the first is: "What quantity of metal is requisite for a lightning rod?" and the following observations are to the point:

"Perhaps one of the most terrific discharges of lightning ever experienced, was that in the case of the New-York packet, struck by lightning in the Gulf Stream, in April 1827; already noticed. In this case the discharge fell on a pointed iron rod four feet long, and half an inch in diameter; some few inches only of the rod near its point were melted; the linked iron chain which descended from this rod to the water, about a quarter of an inch in diameter, was knocked in pieces by the expansive force of the shock, and some of the links fused. The flash of lightning not only melted some of the links, but "caused them to burn like a taper." "The melted iron fell in glowing drops upon the deck, which was instantly set on fire wherever the burning matter fell." "Such was the violence of the shock, that the ship recoiled, or, in sea phrase, lurched so strongly as to throw down the people on deck." The results of a great natural experiment are here presented to us, and we see that an iron rod of half an inch in diameter, effectually resisted a flash which fused and destroyed a chain of about one half of its dimensions.

The flash of lightning which struck Her Majesty's ship *Rodney*, in December 1838, was so powerful that it quite dispersed the topgallant-mast in small chips, covering the sea with splinters, set fire to the main-topsail, tore a piece ten feet long out of the topmast, burst thirteen hoops on the mainmast, each five inches wide and half an inch thick, and traversed the mast with destructive effect for fifty-thee feet. Now this charge passed without fusion on the copper funnel, sixteen inches long, ten inchs in diameter, and less than a quarter of an inch thick, belonging to the topgallant rigging.

In September 1833, two successive discharges of lightning fell on His Majesty's ship *Hyacinth*, in the Indian Ocean, and descended by the fore and mainmasts to the sea. The topgallant and topmasts were literally shaken into a bundle of laths, so that they could scarcely be supported; the topmasts thus shivered were from eleven to twelve ieehes in diameter and about forty feet in length. This destructive shock was safely conducted away from the termination of the topmasts by a chain sheet fifty feet long, made of iron rod half an inch in diameter, and finally through the ship by a copper pipe, three inches in diameter, one-eleventh of an inch thick, and ten feet long; before reaching these bodies, which were direct conductors to the sea, the discharge ravaged and destroyed the masts through a distance of nearly eighty feet; the chain did not show any marks of fusion.

In the summer of the year 1760, a heavy discharge fell on a conductor fixed on a house in Philadelphia. This conductor consisted of an iron rod, half an inch in diameter; it extended nine feet above the chimneys, and terminated in an iron stake in the ground. The part above the chimneys was tipped with brass rod, about a quarter of an inch in diameter, and ten inches long. Mr. West, the owner of the house, judging from the crash that the conductor had been struck, had it examined. About three inches of the brass rod were observed to have been fused, and some of the fused metal had sunk down about the remaining part of the rod, forming a rough, irregular cap about it. The house was not damaged, nor were any further effects produced on the conductor.

On the 26th of January, 1838, a flash of lightning fell on Her Majesty's ship *Dublin*, of fifty guns, at Rio de Janeiro. It was carried off by a conductor of long copper links, each link being about a quarter of an inch in diameter, and ten inches long. The conductor was "regularly melted" in several parts; these fell on the deck; other parts, which remained attached to the line supporting the chain along the rigging of the ship, appeared as if they had been exposed to a very fierce heat. This line was not hurt, nor was any damage done to the rigging.

In June, 1772, a discharge of lightning fell on the Vicarage house at Steeple Ashton, in Wiltshire. The iron bell-wires in both the parlors and in the hall were entirely dispersed, *except in their twisted or double portions*.

In June, 1828, a heavy flash of lightning fell on the spire of Kingsbridge church, in Devonshire, and was received on an iron spindle, seven feet long, and one inch in diameter, without producing the least effect on it, or damaging the stones around it; but on leaving the spindle, however, it shattered the tower, and did considerable damage.

In the case of Charles church at Plymouth, although the wire was knocked in pieces at the points of junction, and considerably bent, it was not materially damaged by fusion.

In reviewing, in this way, a great variety of cases in which buildings and ships have been exposed to appalling storms of lightning in various parts of the world, and throughout a century of years, we are enabled to appreciate the power of metallic bodies to carry off lightning with safety. Now we do not find in any of these cases that a conducting rod, or other mass of metal, equal in substance or conducting power to a rod of copper half an inch in diameter and six inches long, has ever been fairly melted. On the contrary, heavy discharges have traversed rods of less dimensions with safety.

Besides, the conducting power of lightning rods has been much increased of late by improvements in their construction. 1st. In multiplying the number of their points. 2d. In perfecting their continuity. 3d. In the substitution of square rods for round ones. The reasons of these facts will appear hereafter. We will here stop only to add, what Mr. Harris, F. R. S., in his work on thunderstorms, affirms, viz, 'when an acutely terminated lightning rod receives a charge of lightning, a very considerable portion of the charge, if not the whole, runs off in an attenuated stream.' "

The facts upon the range of "the protecting power of the lightning rod" are of great value, and the want of knowledge of these facts has led to many errors, and to consequent disasters. The reader will be well instructed in reading the following observations:

"It is not easy to assign the limit of the protecting power of a conductor. The French philosophers consider it will afford protection over a circle equal to twice its radius; this, although possible in certain cases, is by no means a general truth. All the experience we have of the operation of conductors on discharges of lightning, tends to the conclusion, that they have no influence whatever in determining the course of such discharges, farther than arises out of the circumstance of their furnishing an easy line of conduction. That they do not always afford protection over any considerable distance, is clear from the following cases:—

Her Majesty's ship *Endymion*, commanded by Captain the Honorable F. Gray, was struck by lightning at Calcutta, in March, 1842. This frigate had a chain conductor on the mainmast, applied in the usual way, not very dissimilar to that recommended in the report of M. Gay Lussac to the Royal Academy of Sciences at Paris. The electrical explosion, instead of falling on the conductor, struck the foremast, shivered the top-gallant and topmast, and damaged the lower mast. Now, in this case, the mast struck was not above fifty feet distant from the mainmast, which was furnished with a conductor, and had a radius of one hundred and fifty feet.

Her Majesty's ship *Ætna* was struck by several heavy electrical discharges at Corfu, in January, 1830. These, for the most part, passed down a chain conductor attached to the mainmast. One of the discharges, however, struck the ship near the bow, and exploded about twelve feet above the forecastle, close to the foremast, knocking down all the people on deck, and doing other damage.

The Board-house, at Purfleet, was struck by lightning on the 13th of May, 1777, at a point upwards of forty feet from the conductor with which the house was furnished. The damage, it is true, was small; a few stones fastened by iron cramps not connected with the conductor were thrown down. The Board-house was a lofty building, with a pointed roof, well leaded, and connected by lead gutters and pipes with the earth, and with wells forty feet deep, for the purpose of conveying water forced up to a cistern in the roof. It was therefore only thought necessary to add an iron spike, about ten feet long, to the middle of the highest part of the roof. About one hundred and fifty yards from this building were five powder magazines, each one hundred and sixty feet long and fifty-two feet wide, having spiked conductors at each end projecting ten feet above the roofs, and connected with wells of water. It is quite apparent here, that so far as relates to the *influence* of a conductor over a given area, the experiment is conclusive, and the result shows that we cannot always calculate on the radius of protection; thus confirming the deductions already arrived at.

The Poor-house at Heckingham was struck by lightning on the 17th of June, 1781, which damaged one of the extreme corners of the building, situated seventy feet from the pointed conductors with which the house was furnished. Little or no damage, however, was sustained. The house consisted of a central range of buildings and two flanks; in general form, approaching that of the letter H. There were eight chimneys; each had a pointed conductor. The flash appears to have divided in this case before reaching the ground. One portion struck on one of the conductors, and was carried off; a second struck the extreme point of the building, and set it on fire; a third fell on the earth immediately in front of it.

The house at Tenterton, already referred to, had two stacks of chimneys at each end. To one of these stacks was fixed a conductor of iron rod, projecting five feet above the chimney. Now, the discharge fell on one of the chimneys fifty feet distant, at the opposite end of the building; being the chimney diagonally opposite to that on which the conductor was placed, and on passing to the earth, it did considerable damage. The whole of this shock was finally concentrated on an iron bar three-quarters of an inch square, and produced no effect on it as already noticed. The house was about thirty feet wide."

In section third, the following points are investigated with ability, and the conclusions are reached with clearness: 1st. "Whether lightning rods and other metallic conductors have effectually guarded buildings, &c., against damage by lightning." 2d. "Whether lightning rods attract lightning." 3d. "Whether in certain cases pointed conductors actually prevent explosions of lightning from falling on buildings." Upon all these queries the facts gathered in this treatise are full of interest, and bear directly on the question discussed. The remaining points of interest in these investigations are: "Phenomena observed when a dense explosion of electricity falls on a lightning rod;" "Harmless character of the luminous appearances observed on lightning rods;" "Division of the charge;" "Instances in which buildings provided with pointed conductors are said to have been damaged by lightning;" "Precautions when exposed to thunderstorms;" and "Construction of lightning rods applied to buildings." There is a large amount of valuable, instructing and interesting matter under these various heads, which we should be pleased to quote, but our waning space admonishes us to restrict our extracts to the important point of Practical deductions from the facts and principles. We begin with Mr. Morgan's very perfect plan of securing buildings against damage by lightning.

"The plan which Mr. Morgan proposes is, that, whilst a house is being built, 'the foundation of each partition wall should be laid on a strip of lead, or a strip should be fastened to the sides of these partion walls. The strips should be two inches wide, and at least a quarter of an inch thick, and closely connected with each other. A perpendicular strip, on each side of the house, should rise from the conductors to the surface of the ground; whence a strip should be continued round the house, and carefully connected with water-pipes, &c. The strips on the sides of the house should then be continued to the roof, which ought to be guarded in the same manner as the foundation. The top should be surrounded by a strip, which should be connected with every edge and prominence, and continued to the summit of each separate chimney.' It is particularly necessary to guard the chimneys; for Mr. Morgan mentions a case in which a house that had been guarded in most respects, according to the preceding directions, except that the chimneys were unprotected, was struck with lightning, which entered by one of the chimneys; here it spent its fury; but the chimneys falling on the roof di[illegible] considerable damage.

All electricians agree 'that security is rendered more perfect by having every piece of metal on the roof in metallic connection with the conductor, and continuous strips of lead built into every wall and connected to one another by horizontal strips communicating also with the conductor.'

The only objection to this method of protection is the expense. But even this is mainly obviated, by having the conductors so constructed as to run the whole length of the building on the ridge, with branches to the chimneys, and duly elevated above them; and, in case of small buildings, continuing the main rod on the ridge over the roof, down the opposite diagonal corners of the building to the ground; and if the building is large, by having rods extend from the main rod on the ridge over each end of the roof, down the four corners; and having all the conductors united to one another by a perfect metallic union."

The principles that are reliable in an effort to secure any object to be protected from damage by lightning are thus announced in Mr. Lyon's treatise:

"1. The conductor should be made of good conducting substance.

2. It should have great electrical capacities; a square rod requires less metal than a round rod.

3. It should be perfectly continuous, *i. e.*, it should have no breaks in the connection; no links or hooks, but a perfect metallic union of every part. If a person should think that a ring of zinc near these connections, improves the appearance of the rod, there can be no objection, though there is no other possible advantage.

4. It should be insulated from the building to be protected, except from such masses of metal as are likely to offer other lines of discharge.

5. It should have numerous lateral points:—one in six or seven feet will answer. The more numerous these points are, the greater the condu ting power of the rod. Besides, these lateral points provide for an oblique discharge, each being as good a receiving point as the higher point at the chimney or other prominences. They also guard against a lateral explosion, or a division of the charge, which is liable to happen in case the rod is overcharged, especially if it be fastened to the house with pointed staples; and in case of an upward stroke, the electric fluid being discharged at so many different points, no harm can possibly occur.

6. Its upper extremity should project freely into the air; should be pointed; and may be triangular, somewhat similar to a bayonet; or it may have several branches. The only scientific advantage in having a branching head or point for the superior termination, is this: all the points are not likely to become blunt at the same time. Some have supposed that the point should be *magnetized;* and little needles, called "*magnets,*" have sometimes been added. But is is difficult to see the practicability of this recent discovery; for most are aware, that magnetized iron or steel soon looses its magnetic influence. But, *is* there any truth or science in this application of magnetism? If there *is*, we confess, that we have not been able to discover it in any experiment in the laboratory; neither can we learn that the subject has even been *mentioned* by any writer *whatever*, on the subject of electricity.

7. The upper termination should be plated with silver or gold, to prevent corrosion.

8. Every branch rod running to chimneys, and other prominences, should have a perfect metallic union with the main rod.

9. In cases where metallic vane-spindles, or other points exist, the conductor may commence from these, and should be applied immediately to the part to be protected, and not at a distance from it; and should be so applied, that a discharge of lightning falling on the general mass, could not possibly find its way to the ground through the building by any circuit of which the conductor did not form a part; that is to say, the conductor should be so carried over the several parts of the building, that the discharge could not fall upon it without being transmitted safely by the conductor. Hence, the rod should run along the whole length of the ridge, and down to the ground, at least on two sides of the building. If the building is large, it should run down on each corner.

10. Every conductor running to the ground, should terminate sufficiently beneath the surface, to insure moisture in the dryest part of the season. If circumstances permit, it should connect with a spring of water, a drain, or some other conducting channel."

We have thus endeavored to point out the principal elements of protection against the ravages of lightning, and medical men should endeavor not only to be able to advise correctly when consulted on this subject, but should be prepared to interfere when they see palpable blunders committed in an effort to disarm electricity of its terrible power. It must strike the reader that among the sources of success, there are none more important than a well made rod, securely united at the joints, perfectly insulated, and properly pointed. We have never seen any rod that so perfectly fulfilled these conditions as that made by Spratt. That conductor has no imperfections, and if it is put up according to the laws expounded in Lyon's treatise and quoted in this review, the protection may be regarded as very perfect.

We cordially thank Mr. Lyon for his seasonable treatise.

